# Increased Plasma Cardiac Troponin I in Live-Stranded Cetaceans: Correlation with Pathological Findings of Acute Cardiac Injury

**DOI:** 10.1038/s41598-020-58497-3

**Published:** 2020-01-31

**Authors:** Nakita Câmara, Eva Sierra, Antonio Fernández, Manuel Arbelo, Marisa Andrada, Antonio Espinosa de los Monteros, Pedro Herráez

**Affiliations:** Veterinary Histology and Pathology. Institute of Animal Health and Food Safety (IUSA). Veterinary School. University of Las Palmas de Gran Canaria, Las Palmas de Gran Canaria, Spain

**Keywords:** Cardiomyopathies, Diagnostic markers

## Abstract

Capture myopathy (CM), is a syndrome that occurs as the result of the stress during and after capture, handling, restraint, and transport of wild animals. Although CM has been described for many species of cetaceans, characterization of the acute cardiac injury - an important component of this syndrome - are still scarce. In this study, we firstly estimated a normal range for cardiac troponin I (cTnI) on cetaceans. Here, through biochemical analysis (especially of cTnI) and histopathological, histochemical, and immunohistochemical correlations with decreased troponin immunolabelling, we studied the cardiac injury in live-stranded cetaceans. Nine cetaceans which stranded alive on the Canary Islands (January 2016 - June 2019) were included in this study. Sampled individuals presented elevated values of plasma cTnI, which were correlated to histopathological lesions comprised of vascular changes and acute degenerative lesions. Immunohistochemically, injured cardiomyocytes showed a decreased intrafibrillar troponin immunoreaction. This is the first attempt to establish a normal baseline range for cTnI in cetaceans, and the first study comparing plasma biomarkers values with histopathological and immunohistochemical findings. This approach allowed us to demonstrate the degree of cardiac damage as a result of injury, consistent with ischemia–reperfusion lesions. The knowledge gained here could improve decision-making procedures during stressful situations, mainly in live-strandings, handling, and rehabilitation, thereby reducing the mortality of cetaceans.

## Introduction

All life forms have evolved mechanisms to cope with stressful situations in their lives^1^. For cetaceans, live-stranding is a situation in which they are alive on the beach or in shallow water, and in distress due to being unable to free themselves and resume normal activity^2–4^. Similar to humans and other animals, once the central nervous system of a cetacean perceives an internal (physiologic or psychogenic) or environmental threat to their homeostasis, the threat being the stressor, a biological response is developed^5–7^.

Irrespective of the animal’s previous health, stranding creates an anomalous and extreme situation for an organism that it is not anatomically or physiologically adapted to handle. It is a pathological entity in which acute stress is the central axis of its etiopathogenesis, presenting clinical and lesional findings that can cause the death of the animal or seriously aggravate a previous disease over the period of capture or captivity. This reaction may in turn influence the subsequent rehabilitation and recovery of affected animals, since live-stranded cetaceans are frequently debilitated when rescued. Therefore, although the stranding response is intended to improving the health and welfare of these animals, some “rescue and recovery” activities may in fact be counterproductive^4,8–13^.

Deaths of live-stranded cetaceans may be attributed to the so-called “stress response syndrome” or “alarm reaction,” which are thought to have analogous mechanisms to capture myopathy (CM), in which cardiac damage due to the extreme stress seems to play an important role^2,8,10–12,14,15^. For this reason, previous authors have suggested that cetaceans would be especially predisposed to develop stress cardiomyopathy (SCMP), comparable to SCMP in humans which appears to be related to an excess of plasma catecholamines triggered by a stressful event^2,8,10–12,14–27^. There are no previous studies regarding biochemical cardiac markers that indicate damage to the heart of marine mammals (more specifically in cetaceans).

When irreversible cardiac damage occurs, troponin is released from cardiomyocytes. Troponin is a regulatory protein complex comprised three subunits; troponin C, the calcium binding component; troponin T, the binding component tropomyosin; and troponin I, the inhibitory component. All components are involved in the contractile process of both skeletal and cardiac muscle. The troponin C expressed in skeletal muscle is identical to cardiac troponin C (cTnC). However, cardiac troponin T (cTnT) and cardiac troponin I (cTnI) are specific to the heart^28^.

Very low amounts of cTnI are detectable in the blood of healthy human individuals with no evidence of cardiac disease, as opposed to the amount of cardiac troponin released from cardiomyocytes into the blood as a result of injury, which is consistent with ischemia and other causes^29–31^. Due to this specificity, cTnT and cTnI are recommended by various international societies as a diagnostic indicator for acute myocardial infarction (AMI) and other heart pathologies, such as SCMP, since they are potential markers of cardiac damage^32^.

The histology that defines CM consists of ischemia-reperfusion injuries consistent with local to generalised vasospasm and vasodilation (catecholamines, neurogenic shock, and impeded venous flow return from body compression, causing hypoxia in different organs), direct traumatic injury to the muscle resulting in acute to subacute degeneration (rhabdomyolysis), acute renal failure associated with myoglobinuric nephrosis, and areas of necrosis in viscera^9–11,13–15,33–35^.

The aim of this study was to estimate a normal range for cTnI in cetaceans and describe the acute cardiac injury in live-stranded cetaceans, correlating biochemical analysis with histological, histochemical, and immunohistochemical findings.

## Results

The reference range of cTnI was of 0–0.0256 µg/L, determined with a 95% probability. All data necessary to calculate this range are shown in Supplemental Table 1.

**Table 1:** Summary of the stranding circumstances, biochemical results, histopathological findings and immunohistochemical changes for each animal of the study.

			Animal 1 (^*^)	Animal 2 (^†^)	Animal 3 (^‡^)	Animal 4 (^§^)	Animal 5 (^‡^)	Animal 6 (^§^)	Animal 7 (^‡^)	Animal 8 (^||^)	Animal 9 (^*^)
			*Delphinus delphis*	*Delphinus delphis*	*Stenella coeruleoalba*	*Stenella coeruleoalba*	*Globicephala macrorhynchus*	*Delphinus delphis*	*Stenella coeruleoalba*	*Stenella coeruleoalba*	*Stenella coeruleoalba*
STRANDING CIRCUNSTANCES	TIME OF STRANDING NOTICE		10:16 AM	11:25 AM	5:12 PM	11:13 AM	11:57 AM	9:30 AM	8:37 AM	9:38 AM	12:35 PM
	TIME OF DEATH		12:00 PM	12:00 PM	6:45 PM	NO	12:00 PM	NO	8:45 AM	NO	2:38 PM
	TIME OF SAMPLE COLLECTION		10:00 PM	4:00 PM	6:45 PM	11:47 AM	3:37 PM	10:00 AM next day	11:00 AM	2:00 PM	3:28 PM
BIOCHEMICAL ANALYSIS	TROPONIN I (cTnI) (µg/L)	*Animal Value*	0.06	40.00	0.025	0.235	0.249	0.748	0.033	0.06	0.049
		*Baseline Range Cetaceans*	0–0.0256 (^#^)								
		*Baseline Range Dogs*	≤0.03–0.07 (^**^)								
		*Baseline Range Humans*	≤0.1 (^**^)								
	CREATININE KINASE (CK) (U/L)	*Animal Value*	1875.0	Not measured	1667.1	Not measured	3521.2	3299.5	383.6	Not measured	739.2
		*Baseline Range*	100–250 (^††^)								
HISTOPATHOLOGICAL & HISTOCHEMICAL ANALYSIS	VASCULAR CHANGES	*Congestion*	Absent	Mild	No necropsy	Mild to moderate	Mild	Mild	Absent	No necropsy	Mild
		*Interstitial Oedema*	Absent	Absent		Mild to moderate	Mild	Absent	Absent		Absent
		*Haemorrhages*	Absent	Mild		Absent	Absent	Absent	Absent		Absent
	ACUTE DEGENERATIVE CHANGES	*Contraction Band Necrosis*	Absent	Absent		Absent	Mild to moderate	Absent	Absent		Absent
		*Wavy Fibers*	Mild	Absent		Absent	Mild to moderate	Moderate	Absent		Absent
		*Hypereosinophilia*	Mild	Moderate		Moderate to severe	Mild to moderate	Moderate	Mild		Moderate
		*Cytoplasmic Vacuolization*	Mild	Moderate		Moderate to severe	Mild to moderate	Moderate	Mild		Moderate
	INFLAMMATORY INFILTRATION		Moderate	Absent		Absent	Absent	Absent	Absent		Absent
	MYOGLOBIN GLOBULES		Absent	Absent		Absent	Present	Present	Absent		Absent
IMMUNOHISTO-CHEMICAL ANALYSIS	MYOGLOBIN		Frozen animal	Depletion	No necropsy	Depletion	Depletion	Depletion	Frozen animal	No necropsy	Depletion
	CARDIAC TROPONIN I (cTnI)			Depletion		Depletion	Depletion	Depletion			Depletion
	CARDIAC TROPONIN C (cTnC)			Depletion		Depletion	Depletion	Depletion			Depletion
	FIBRINOGEN			Deposition		Deposition	Deposition	Deposition			Deposition

(*) Live stranded animal that dies during transport. (^†^) Live stranding notification (09:00 PM) of a cetacean swimming very close to shore being reintroduced in to sea several times by general public until he swims away. New communication of stranded animal one day and a half after the first notification (11:25). The animal is euthanized. (^‡^) Live stranded animal that died before being attended. (^§^) Dead animal which presented injuries that indicates that the animal stranded alive. (||) Live stranded animal and released back into the sea. (^#^) Baseline range determined in this study. Comparing the interval of reference for cTnI in bottlenose dolphins with the normal values for humans and dogs we can conclude that is shorter than in other species. (**) Values from literature^41–43^. (^††^) Reference range for Tursiops truncatus in captivity^4^.

The serum values of cTnI and creatine kinase (CK) obtained for each animal are shown in Table 1. Additionally, it summarises the evaluation of the vascular changes, acute degenerative changes, and the presence of inflammatory cells. It also shows the presence or absence of myoglobin globules.

**Figure 1:**
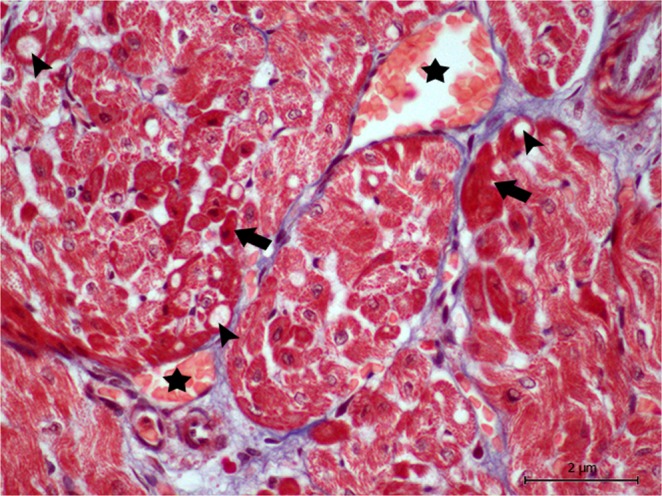
Animal 4. Groups of damaged cardiomyocytes near the blood vessels (*) present a higher staining (arrows), in this case due to Masson’s trichrome, compared to normal cardiomyocytes, which stain less. Note the cytoplasmic vacuolisation (arrow heads). Magnification 60 × .

Regarding animal 1, the biochemical analysis presented 0.06 µg/L for cTnI and 1875.0 U/L for CK. With reference to the histopathological results, we observed a moderate epicarditis, neuritis, and multifocal lymphoplasmacytic perineuritis, and a mild multifocal lymphoplasmacytic myocarditis. We also detected moderate multifocal myocardial fibrosis and mild multifocal acute degenerative changes in the cardiomyocytes consistent with wavy fibres, hypereosinophilia, and cytoplasmic vacuolisation (Fig. 1). We did not assess the depletion or expression of the immunohistochemical markers because the sample was frozen, and previous studies have shown that animals with advanced autolysis or that were previously frozen presented a greater number of false negatives when using histochemical and immunohistochemical techniques^15^.

Biochemically, due to serum insufficiency, the only metric we were able to obtain from animal 2, was a cTnI level of 40.00 µg/L. The histopathology revealed a mild, multifocal congestion and haemorrhages. Moreover, it also showed moderate, multifocal acute cardiomyodegenerative lesions indicated as hypereosinophilia and cytoplasmic vacuolisation. Immunohistochemically, we verified homogenous, intrafibrillar depletion of cTnI, cTnC, and myoglobin in the degenerated/necrotic cardiomyocytes. Furthermore, damaged cells from the cardiac muscle, showed various intensities of immunolabeling for fibrinogen.

The biochemical analysis of animal 3, it presented 0.025 µg/L of cTnI and 1667.1 U/L of CK. We were incapable to obtain histopathological and immunohistochemical parameters, as we were unable to perform a necropsy on this animal, since it was used for anatomical research purposes.

Biochemically, due to serum insufficiency, the only metric we were able to obtain from animal 4, was a cTnI level of 0.235 µg/L. The histopathology revealed mild to moderate, multifocal congestion, and interstitial oedema. Additionally, we observed moderate to severe multifocal acute degenerative changes in the cardiomyocytes, such as hypereosinophilia and cytoplasmic vacuolisation. Immunohistochemically, we detected fibrinogen expression and the disappearance of immunolabeling for cTnI, cTnC, and myoglobin in the degenerated/necrotic cardiomyocytes.

**Figure 2:**
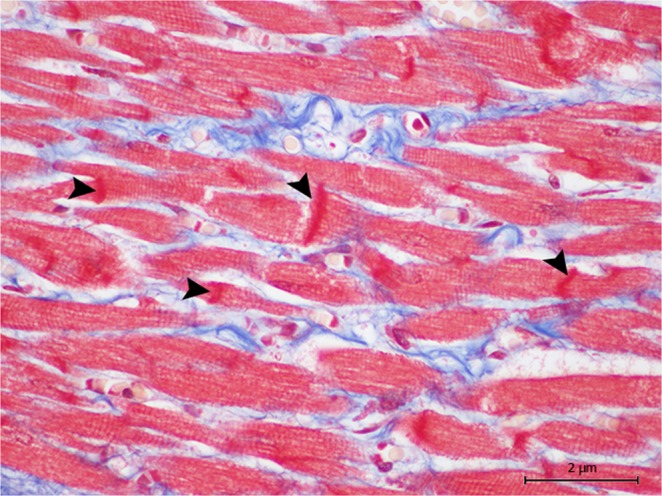
Animal 5. The contraction band necrosis runs (arrow heads) transversely through the cardiomyocytes and is identified through the higher colour intensity with Masson’s trichrome. Magnification 60 × .

Regarding animal 5, the biochemical analysis showed a cTnI level of 0.249 µg/L and a CK level of 3521.2 U/L. The histopathology revealed mild to moderate acute cardiomyodegenerative lesions, consistent with contraction band necrosis (Fig. 2), wavy fibres, hypereosinophilia, and cytoplasmic vacuolisation. Simultaneously, we also verified myoglobin globules and mild, multifocal congestion, and interstitial oedema. The immunohistochemical analysis showed the homogenous intrafibrillar depletion of cTnI, cTnC, and myoglobin in the degenerated/necrotic cardiomyocytes; these also exhibited various intensities of immunolabeling for fibrinogen.

The biochemical results for animal 6 showed 0.748 µg/L for cTnI and 3299.5 U/L for CK. The histopathology showed moderate multifocal myocardial fibrosis and acute degenerative changes in the cardiomyocytes, reflected as wavy fibres, hypereosinophilia, and cytoplasmic vacuolisation. Furthermore, we detected the presence of myoglobin globules and mild multifocal congestion. The immunohistochemical parameters showed the expression of fibrinogen and the disappearance of immunolabeling for cTnI, cTnC, and myoglobin in the degenerated/necrotic cardiomyocytes.

Biochemically animal 7 showed 0.033 µg/L for cTnI and 383.6 U/L for CK. The histopathology showed mild acute cardiomyodegenerative lesions, such as hypereosinophilia and cytoplasmic vacuolisation. We did not analyse depletion or expression of immunohistochemical marker because the sample was frozen.

For animal 8, serum insufficiency prevented us from obtaining all biochemical metrics. We were only able to obtain the cTnI level, which was 0.06 µg/L. We were unable perform histopathology and immunohistochemistry because we were unable to perform a necropsy on this animal as it was released back into the sea.

Biochemically, animal 9 exhibited 0.049 µg/dL for cTnI and 739.2 U/L for CK. The histopathology showed a mild multifocal congestion and moderate, multifocal acute degenerative changes in the cardiomyocytes, such as hypereosinophilia and cytoplasmic vacuolisation. Regarding the immunohistochemistry, we observed homogenous intrafibrillar depletion of cTnI, cTnC, and myoglobin in the degenerated/necrotic cardiomyocytes; we also detected various intensities of immunolabeling for fibrinogen.

## Discussion

Recent studies have characterized cardiac lesions associated with live-stranding in cetaceans. The etiopathogenesis of this acute cardiac pathology has as its central axis the stress derived from stranding, handing, interaction with humans, transport and captivity (having been compared with the CM described in terrestrial mammals). Etiological and lesionally, the acute cardiomyopathy associated with live-stranding is comparable to SCMP in humans. SCMP lesions in live-stranded cetaceans consist of vascular changes (congestion, interstitial edema and hemorrhages) and acute necrotic degenerative lesions (contraction band necrosis, wavy fibers, hypereosinophilia and cytoplasm vacuolization) of perivascular distribution, indicative of ischemia-reperfusion damage^10–12,15,35^.

In humans, the diagnostic criteria for SCMP comprises alterations in medical exams (such as electrocardiography, echocardiography, cardiac catheterizations), and biochemical analysis. The more common laboratories abnormalities are consistent with a small, rapid rise to above the normal levels of cTnI and/or CK^36–38^.

Currently, there are no hematological and/or biochemical studies on cetaceans that analyze specific markers to detect acute heart damage associated with the live-stranding.

The most commonly used serum enzyme in the determination of neuromuscular damage in animals is CK; it is also used in the detection of myocardial injury in humans^30,39^. CK rises less than 3–12 h after the muscular injury, spiking within 12–24 h and returning to baseline after 48–72 h, unless a new injury or permanent damage has occurred^30^. As verified by the present study, and in comparison with the publish literature, CK values were high for all animals we were able to measure it (animals 1, 3, 5, 6, 7, and 9).

However, although CK is considered a sensitive marker of myocardial damage, it is also present in the skeletal muscles in high concentrations, as well as in the intestine, diaphragm, uterus, and prostate in minor amounts; thus, it has poor specificity when used to detect heart damage^40^. Consequently, troponins have been adopted as the new gold standard in human pathology, since cTnI is detectable in very low quantities (for example 0.01 µg/L) in the blood of healthy individuals with no evidence of cardiac disease^29–32^. Therefore, it is thought that significant elevations (≥0.1 µg/L) of this marker most likely reflect myocardial necrosis, and has been billed as cardiospecific by some authors due to its myocardial tissue specificity, as well as its high sensitivity^30–32^. For this reason, cTnI is used to detect several heart pathologies, such as AMI and SCMP^30,36,40^.

Clinical-pathological data evaluation was challenging because values from different species of cetaceans are scarce. Considering this, and because there is no normal range for cTnI in cetaceans to date, we decided that it was important to determine a range of baseline values in order to compare the normal interval for the bottlenose dolphin with the normal parameters of other species, such as humans and dogs, as well as to assess the results of the live-stranded animals in this study.

Comparing the range in this particular study (0–0.0256 µg/L) with the normal values for humans ( ≤ 0.1 µg/L) and dogs ( ≤ 0.03–0.07 µg/L), we can conclude that the interval of reference for cTnI in bottlenose dolphins is shorter than in other species^41–43^. This could be justified by the small number of samples (20 blood samples in total). Nonetheless, we consider this result to be an important contribution to clinical biochemistry in cetaceans, as it may help in decision-making and treatment procedures during stressful situations, such as live-strandings, and improve conservation efforts by reducing the mortality of these animals.

Our results, from the live-stranded cetaceans, showed that one of the animals presented a cTnI value within normal range whilst the remaining eight animals demonstrated an increase in comparison to the normal/baseline value for cTnI (0–0.0256 µg/L) that we obtained from captive bottlenose dolphins. However, when comparing these with those for humans ( ≤ 0.1 µg/L) and dogs ( ≤ 0.03–0.07 µg/L), we observed that animal 1 (0.06 µg/L), animal 3 (0.025 µg/L), animal 7 (0.033 µg/L), animal 8 (0.06 µg/L), and animal 9 (0.049) are within range, while animal 2 (40.00 µg/L), animal 4 (0.235 µg/L), animal 5 (0.249 µg/L), and animal 6 (0.748 µg/L) exceed the upper value^41–43^.

More than 90% of patients with SCMP revealed raised cTnI when measured by conventional assays^36^. However, SCMP is a disease with a high rate of misdiagnosis because it is an important differential diagnosis of an AMI since highly similar clinical presentations occur between them^25,26^. Besides, it is under-recognized because of the unnecessary delay in the diagnosis process in post-mortem examination cases of AMI caused by the less sensitive conventional markers.

Previous authors have stated the necessity of establishing diagnostic biochemical cardiac markers for the post-mortem diagnosis of AMI and SCMP due to the limitations of histopathology. The sensitivity of cTnI makes early detection of microinfarction possible after the onset of ischemia using a rapid one-step assay in body fluids in autopsy cases^40^. Therefore, we selected cTnI as our marker of choice to detect myocardial damage in live-stranded cetaceans, through biochemical and immunohistochemical detection, on the grounds of its specificity, as well as being highly sensitive^30,40^.

Generally, injured cardiac myocytes release troponin 3–9 h after ischemic damage, peaking after 12–48 h and levels remain elevated for 4–7 days for cTnI^30,40,44^. Early recognition of myocardial necrosis (1–3 h) is not possible by monitoring kinetics, and these markers are ineffective until 6 or more hours after the onset of the AMI and/or SCMP. The precise determination of the timing of the stress event and/or symptom onset is often exceptionally difficult because it is focused around the clinical patient report. Therefore, in humans, a precondition to obtaining a satisfactory ability to distinguish these pathologies is that blood should be collected 6–9 h after onset^40^. In the case of a live-stranding event, it is also often clinically and pathologically challenging to have the correct timing of the stressful episode, since we are working with wild animals, stranding networks, and the general public. When notified of stranding it is important to acknowledge that the animal could have been stranded recently or could have been stranded for hours before being detected.

Moreover, previous studies have proposed that measuring the level of troponin in the serum can be an important auxiliary method of examining sudden death, since its peak concentration can be related to the degree of injury^40^. For this reason, this finding was confirmed with the histological, histochemical, and immunohistochemical analyses.

An acute ischemic injury, such as the one that occurs in the AMI or SCMP, is determined by morphological alterations, consisting of vascular changes and acute degenerative lesions based on the histological analysis of myocardial tissue obtained from biopsied and post-mortem examined species, mostly humans, but also in cetaceans^15,19,25,26,37,45–54^.

In acute ischemic injuries there is a chronological sequence of changes, which can be observed as early as the first 5 minutes (presence of long, thinned, wavy fibers separated by spaces representing edema, and microvascular congestion, at borders of ischemic myocardium). In the following 15 minutes cell death may start to occur. Early changes of cardiomyocyte coagulation necrosis with nuclear pyknosis, color change, more specifically “brick red change” or cytoplasm hypereosinophilia, focal contraction bands and subtle interstitial edema are evident within 2–3 h. Hypereosinophilia and edema become more pronounced and more easily recognizable after a period of 3–6 h. Subsequently, 6–12 h later, an increased number of neutrophils line up in capillaries as well as accelerated changes and more extensive contraction band necrosis with reperfusion are noticeable. In the next 12-hour period, extravasation into interstitial space of neutrophils happens. Vascular congestion, interstitial edema, and focal areas of haemorrhage are also recognized. Thereafter, the subacute period starts^15,26^.

Considering all the above, we conclude that all the results obtained from the animals of this study were in accordance with the biochemical kinetics, as well as the chronological sequence of histopathological changes in an acute ischemic injury.

Animal 1 died 2 h after the stranding notice and histopathologically presented mild acute degenerative changes, which indicate that they would fit into the 0–3 h time frame, as expected following the histological sequence.

Elevated biomarker values were observed in animal 2. This can be explained by the fact that the notification, of a cetacean swimming very close to shore, was received and after several attempts by members of the public to reintroduce it into the ocean the animal swam away; despite all the effort 38 h later a new alert was received stating that the animal stranded again. Its histopathology analysis showed mild vascular changes and moderate acute cardiomyodegenerative lesions. All of these are detectable between 0–24 h approximately and in agreement with both biochemical kinetics and histopathological sequence previously mentioned.

The biochemical results for animal 3 were near the maximum of the normal range value for cTnI in cetaceans. The animal died 1 h after the stranding notice, nonetheless, we were unable to compare these results with the histopathological lesions since a necropsy could not be performed.

The external injuries in animal 4 indicated that it had stranded alive, but was already dead by the time it was detected at the beach. Although the time of death is unknown, cTnI value was elevated. Histologically, it presented mild to moderate vascular alterations, and moderate to severe acute degenerative changes. This fact was consistent, with the time frame of 0–24 h, as per the histological sequence discussed earlier.

Considering the biochemical kinetics in animal 5, which died 1 h after the notice, we hypothesise that the stressful event took place sometime before we received the communication of the stranding, since the cTnI results were high. The mild vascular changes, and mild to moderate acute cardiomyodegenerative lesions were compatible with the time frame of 0–24 h of the histological sequence.

In spite of the unknown time of death of animal 6, and similarly to animal 4, the cTnI results were high and revealed mild vascular alterations and moderate degree of acute degenerative changes, which were comparable to the ones illustrated in the 0–24 h time frame of the histological sequence described previously.

The biochemical values obtained for animal 7 are consistent with the kinetics, since the animal died minutes after the stranding notice. Histopathologically presented mild acute cardiomyodegenerative lesions complying with the time frame of 0–3 h expectations for an acute ischemic injury sequence.

Regarding animal 8, the values challenged the kinetics, because the blood sample was collected 5 hours after the stranding event. However, as discussed previously, the levels of cTnI increases 3–9 h after the stressful event. In view of the fact that we were able to release the animal back to the sea, no histopathological study could be carried out for this animal.

Finally, the results of animal 9 are in accordance with the biokinetics mentioned above, since the animal died 2 h after notification of the stranding. It also presented a mild degree of vascular alterations and moderate acute degenerative changes, common to the sequence of an acute ischemic injury histology, for 0–3 h time frame.

It is important to highlight that active stranding implies a bias in the cases analysed. This is because most animals tend to strand themselves due to a previous pathological process, which is not always identifiable. Therefore, the histological lesions described previously were, most likely, directly related to the stranding itself but not related to the cause of the stranding. Nevertheless, the live-stranding could negatively influence the pre-existing pathological process in the animal, aggravating it, and contributing to its death.

For example, animal 1 became stranded/died as a result of a *Morbillivirus* infection (unpublished data) and demonstrated characteristic lesions, such as lymphoplasmacytic infiltration, which can be observed in both *Morbillivirus* infection and in an acute ischemic injury such as the one resulting in AMI or SCMP^8,9,19,45,46^. Another example is the presence of fibrosis, both in animal 1 and animal 6, indicating a chronic phase of the cardiomyocytes damage, since connective tissue proliferation dominates the third week until healing is completed after 3 months, which indicates a previous heart damage had occurred prior to the latest stranding^26^.

**Figure 3:**
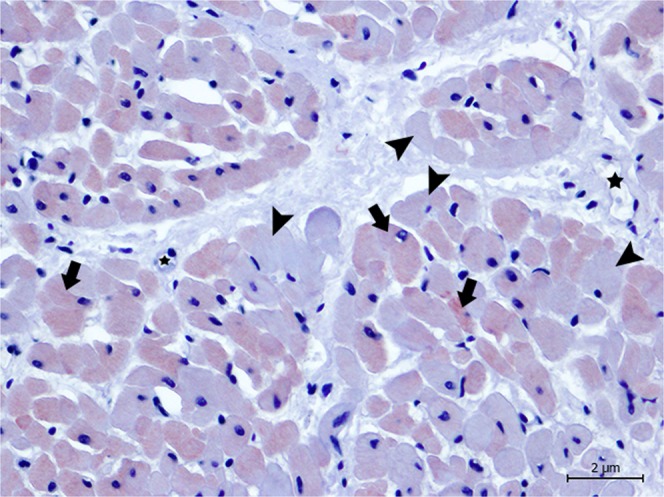
Animal 5. Degenerated/necrotic cardiomyocytes (arrow heads) near the blood vessels (*) present the depletion of cTnI when compared with normal cardiomyocytes (arrows). Magnification 40 × .

**Figure 4:**
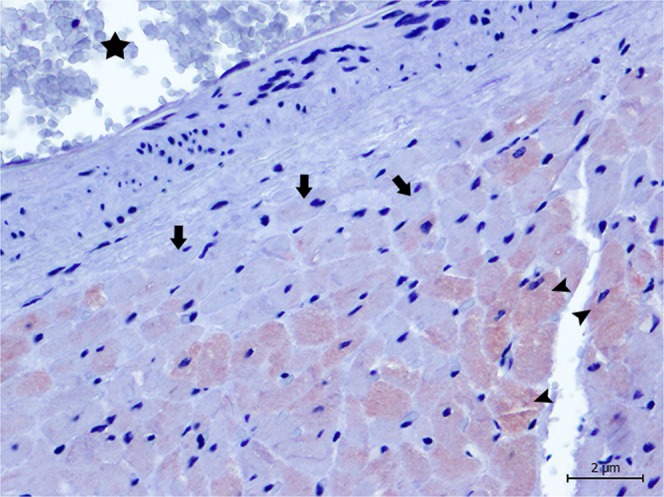
Animal 6. Damaged cardiomyocytes (arrows) reveal a perivascular pattern (*) with decreased immunolabeling for cTnI, in comparison to normal cells (arrow heads). Magnification 40 × .

**Figure 5:**
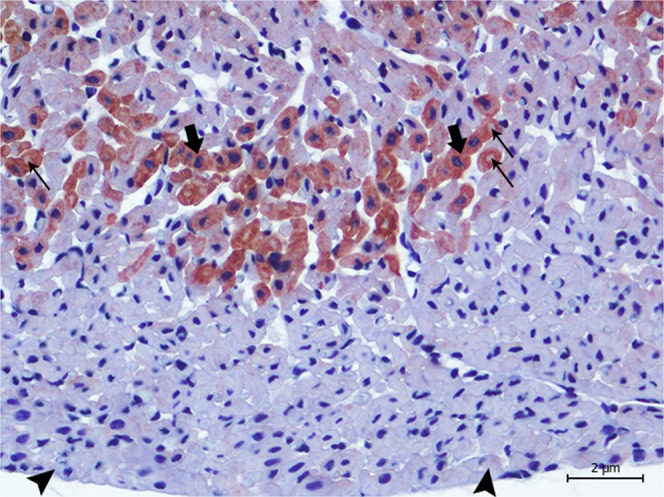
Animal 2. Degenerated/necrotic cardiomyocytes (arrow heads), especially in the epicardium, present the depletion of cTnC when compared to the normal cardiomyocytes (arrows). Note the presence of cytoplasmic vacuolisation (thin arrows). Magnification 40 × .

**Figure 6:**
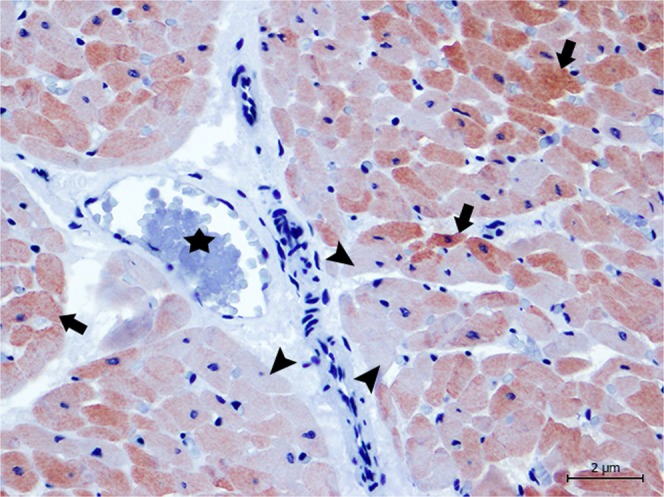
Animal 5. Damaged cardiomyocytes (arrow heads) reveal a perivascular pattern (*) with decreased immunolabeling for cTnC, in comparison to normal cells (arrows). Magnification 40 × .

Previous studies have demonstrated the necessity of corroborating biochemical and histological findings with specific markers that might better delineate myocardial damage^10^, so we compared the markers cTnI, cTnC, myoglobin and fibrinogen^10,11,15,44,55–59^. Immediately after acute ischemia caused by a vital trauma, muscle (skeletal and cardiac) proteins begin to leak as a result of early skeletal and myocardial cell membrane rupture, causing a rapid decline in myoglobin, cTnI, and cTnC contents, along with the deposition of plasma proteins, such as fibrinogen^10,11,15,55–57^. Therefore, damaged cardiomyocytes, in the absence of necrosis during myocardial injury, release cTnI and cTnC, resulting in an increase in serum levels and decreased cardiomyocyte troponin immunoreaction^15,40,60^. In the present study, the amount of damage present in cells was tested using immunohistochemical labelling of the animals that we were able to perform a complete necropsy and had not been previously frozen (animals 2, 4, 5, 6, and 9). All presented tissue depletion, especially cTnI and cTnC (Figs. 3–6) as well as myoglobin, together with intrafibrillar fibrinogen deposition. Consequently, with these immunohistochemical changes, we confirmed that the lesions present in these animals were ante mortem.

In conclusion, although the descriptions of CM has been reported in different species of cetaceans, characterisation of the cardiac damage in marine mammals – which is an important part of this syndrome – are still scarce. It is acknowledged and proven that live-stranding, itself, is an extreme and intensive stressor. Here, we describe the first clinical and pathological study of cardiac injury in live-stranded cetaceans – through biochemical analysis (especially of cTnI) and their histopathological, histochemical, and immunohistochemical correlations with decreased troponins immunolabeling – being the results highly comparable to the existing ones for SCMP in humans. We recommend further studies to advance our understanding and knowledge of the cardiac clinical-pathology of cetaceans.

## Materials and Methods

Firstly, this report determines a baseline range for cardiac troponin I (cTnI), a specific cardiac biomarker for the detection of cardiac damage in cetaceans, more specifically bottlenose dolphins (*Tursiops truncatus*). Secondly, it describes the biochemical analysis and histological, histochemical, and immunohistochemical features of cardiac injuries associated with live-stranding in different species of cetaceans from the infraorder *Odontoceti*.

### Phases and cases of the present study

The first phase of this study consisted in the determination of normal values for cTnI in cetaceans, specifically bottlenose dolphins, with a 95% probability through measurements obtained from captive animals (n = 5) at a local zoo. To achieve this, a total of 20 blood samples were collected, in June, September and December of 2018 and March of 2019 (4 samples of each individual, 1 sample in each month).

In the second phase, a total of 9 animals of four different species, including small and large odontocetes, were included in this study. Blood samples from these animals were collected, being one pre-mortem and the rest post-mortem since the animals subsequently died previous to or during the handling, restraint, transport, and/or rescue/rehabilitation (n = 8). All of the specimens were stranded alive on the coast of the Canary Islands from the beginning of 2016 until June of 2019.

During the third phase, a total of 7 animals were selected, from the animals included (n = 9) in the previous phase, for the histological and histochemical study, since we were not able to perform a necropsy in 2 animals, because one was released back to sea and another was used for anatomical research purposes.

In the fourth and last phase, 2 animals (from the prior phase) were eliminated from the study due to their prior freezing. Therefore, at the end of this phase, a total of 5 animals were studied with the use of the immunohistochemical technique for the detection of different markers.

**Figure 7:**
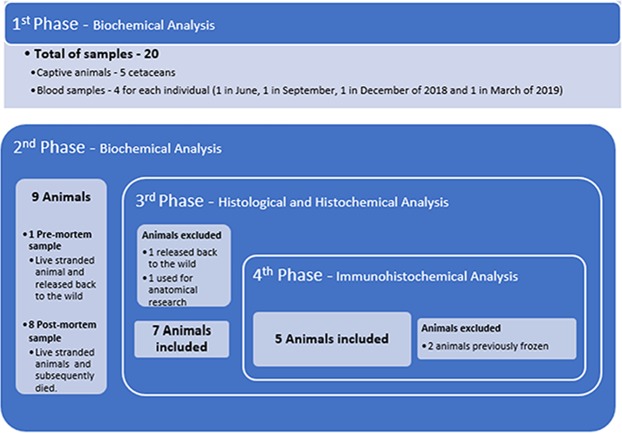
Diagram explaining the different stages of the present study.

In Fig. 7 we present the different stages formerly described. The stranding circumstance, basic and epidemiological data of each animal, included in this study, are detailed in Table 1 and Supplemental Table 2, respectively.

### Biochemical analysis

Whole blood samples for each animal were collected in a gel tube (without anticoagulant), allowed to coagulate, and centrifuged at 3500 rpm for 5 min, two times to obtain the serum (approximately 1 ml).

### Baseline range determination of cTnI in cetaceans

As the sample size is less than 40 individuals, we have had to assume that each of the variables is normally distributed and that for each individual, and for the variable (troponin), the measured value follows a model of the form *x*_*ij*_ = *μ* + *b*_*i*_ + 𝜖_*ij*_, where 𝑖 is the index of the individual (*i* from 1–5) and 𝑗 is the order of measurement within the individual (*j* from 1–4)^61,62^. It has been assumed that the individual effect of the animal is *b*_*i*_ ≈ (0, *σ*_*b*_), and that the variability within each animal is 𝜖 ≈ *N*(0, *σ*_𝜖_), where the values of *σ*_*b*_ and *σ*_𝜖_ are specific to each variable. Under these assumptions, the values of each variable follow normal distributions of the form *X* ≈ (*μ*,√*σ*_𝑏_^2^ + *σ*_𝜖_^2^). The IMER library was used to estimate the parameter, and the anovaVCA library was used to estimate the variances, both using the statistical package R^63^. Once these quantities were estimated, the normality intervals were calculated according to the expression $[\mu -{Z}_{a/2}\surd {{\sigma }_{b}}^{2}+{{\sigma }_{\varepsilon }}^{2},\mu +{Z}_{a/2}\surd {{\sigma }_{b}}^{2}+{{\sigma }_{\varepsilon }}^{2}]$, where *Z*_*α*/2_ is the *α*/2 percentile of the normal distribution. Under all of these assumptions, the normal values of each of the variables considered can be expected to fall within this range with a probability of 1 − *α*. Therefore, we have constructed normal intervals with a 95% probability.

### Biochemical analysis of the live-stranded cetaceans included in this study

On eight animals, blood samples were retrieved 1–38 h after the live-stranding and with a minimum of 2 h to a maximum of 24 h after the death of the animal. Additionally, one sample was recovered pre-mortem and within 5 h of the stressful event. The main biochemical markers analysed were cTnI, which reveals a specific heart injury, and CK, which demonstrates acute muscle damage, both cardiac and skeletal. The results of the biochemical analyses were compared to published values of different mammals, such as humans and domestic animals (dog), in respect to cTnI and diverse cetaceans of the infraorder *Odontoceti* in the case of CK whenever possible.

### Histological and histochemical analysis

Complete necropsies were performed following the standard protocol, with the animals (n = 7) presenting a very fresh or fresh state of decomposition^2,64^. For histopathological analysis, the heart muscle (both atria and ventricles, different atrioventricular valves [bicuspid or mitral and tricuspid], and semilunar valves [sigmoid aortic and pulmonary] with the corresponding arteries) were fixed and processed. Specifically, 4 µm of the samples mentioned above were used for haematoxylin and eosin staining and Masson’s trichrome techniques.

The sections subjected to the histochemical techniques were examined in a blind manner by three veterinarians (NC, ES, and PH). The sections were evaluated for vascular changes (congestion, haemorrhages, and interstitial oedema); acute degenerative changes (contraction band necrosis, wavy fibres, perinuclear vacuolisation, cytoplasmic hypereosinophilia, and pyknotic nuclei), as well as the presence of interstitial myoglobin globules, and infiltration of inflammatory cells. The extent of cardiac lesions was judged subjectively as follows: absent, mild, mild to moderate, moderate, moderate to severe, and severe.

### Immunohistological analysis

Tissue sections of 3 µm, from each of the animals (n = 5), were immunolabeled using specific markers of myocardial injury, such as anti-cTnI, anti-cTnC, anti-myoglobin, and anti-fibrinogen primary antibodies, and were visualised using the VECTASTAIN® Elite ABC-Peroxidase Kit with reference PK-6100 (Vector Laboratories, Peterborough, United Kingdom). The methodology is summarised in Supplemental Table 3. For the immunohistochemical techniques, the positive control for cTnI and cTnC was a heart sample from a pig and a cetacean, respectively, with no apparent acute macroscopic and/or histological lesions, nor a live-stranding history in the case of the cetacean. For myoglobin and fibrinogen, we used a heart sample from a previously published case of a striped dolphin that was stranded alive and developed CM due to capture and the rehabilitation process^10,11^. Finally, the negative control was performed without the primary antibody.

The sections submitted to the immunohistochemical techniques were examined “blind” by three veterinarians (NC, ES, and PH). The amount of cell damage was confirmed as ante-mortem when it was accompanied with the immunohistochemically demonstrated depletion of myoglobin together with intrafibrillar fibrinogen deposition.

### Evidence of ethical approval

Blood collection from the captive animals was carried out by specialised veterinarians authorised by “Palmitos Park” Zoo, within their routine prophylactic program, as well as their use for this scientific study, in compliance with the requirements established on the articles 3, 4 and 5 of the “Ley 31/2003, de 27 de octubre, de conservación de la fauna silvestre en los parques zoológicos” (BOE-A-2003-19800) and the Council Directive 1999/22/EC of 29 March 1999 relating to the keeping of wild animals in zoos (EUR-Lex -31999L0022). Likewise, the use of blood samples for this scientific study was expressly authorised by the Director of the “Palmitos Park” zoo.

Regarding the management of stranded cetaceans, required permission was issued by the environmental department of the Canary Islands’ Government and the Spanish Ministry of Environment.

## Supplementary information

Supplemental Tables.

## Data Availability

All data reported in this work are classified and stored in the tissue bank of the Institute of Animal Health and Food Safety (IUSA). Veterinary School. University of Las Palmas de Gran Canaria.
