# Supplementary material for: Increased Plasma Cardiac Troponin I in Live-Stranded Cetaceans: Correlation with Pathological Findings of Acute Cardiac Injury
Source: Sci Rep. 2020 Jan 31;10:1555. doi: 10.1038/s41598-020-58497-3 (PMC6994679; doi:10.1038/s41598-020-58497-3)
Supplement: Supplementary file 1 — Supplemental Tables. [file 41598_2020_58497_MOESM1_ESM.docx]

**Supplemental Tables of the manuscript:**

**Increased Plasma Cardiac Troponin I in Live-Stranded Cetaceans: Correlation with Pathological Findings of Acute Cardiac Injury**

Nakita Câmara^1^, Eva Sierra^1^*, Antonio Fernández^1^, Manuel Arbelo^1^, Marisa Andrada^1^, Antonio Espinosa de los Monteros^1^, and Pedro Herráez^1^

1 Veterinary Histology and Pathology. Institute of Animal Health and Food Safety (IUSA). Veterinary School. University of Las Palmas de Gran Canaria, Las Palmas de Gran Canaria. Spain

*** Corresponding author:** Institute for Animal Health and Food Safety. Veterinary School. University of Las Palmas de Gran Canaria. Transmontaña s/n. 35416, Arucas, Las Palmas de Gran Canaria, Canary Islands, Spain. ([eva.sierra@ulpgc.es](mailto:eva.sierra@ulpgc.es)) (+34 928 45 97 08).

**Supplemental table 1. Summary of the cTnI values of the different blood samples collected from the 5 Bottlenose dolphins living in the Zoological Park.**

|  | **JUNE 2018** | **SEPTEMBER 2018** | **DECEMBER 2018** | **MARCH 2019** |
| --- | --- | --- | --- | --- |
| **ANIMAL ZP 1** | 0.015 | 0.015 | 0,001 | 0.004 |
| **ANIMAL ZP 2** | 0.015 | 0.015 | 0.003 | 0.001 |
| **ANIMAL ZP 3** | 0.015 | 0.015 | 0.016 | 0.001 |
| **ANIMAL ZP 4** | 0.024 | 0.015 | 0.004 | 0.002 |
| **ANIMAL ZP 5** | 0.015 | 0.015 | 0.001 | 0.002 |

**Supplemental table 2. Summary of the data of the cetaceans participating in the study.**

|  | **INTERNAL ANIMAL CODE** | **SAMPLES CODE (HISTOLOGICAL CODE)** | **SPECIES** | **SEX** | **AGE** | **PRESERVATION STATE** | **STRANDING LOCATION** | **TYPE OF STRANDING** | **PATHOLOGICAL ENTITY / CAUSE OF DEATH** |
| --- | --- | --- | --- | --- | --- | --- | --- | --- | --- |
| **ANIMAL 1 (*)** | CET 810 | (i907/16) | *Delphinus delphis* | Female | Juvenile | Fresh | Tenerife | Live | Pathology associated with significant loss of nutritional status |
| **ANIMAL 2** | CET 860 | (i219/17) | *Delphinus delphis* | Female | Calf | Very Fresh | Lanzarote | Live | Neonatal/Perinatal pathology |
| **ANIMAL 3 (†)** | CET 893 | (SA34/18) | *Stenella coeruleoalba* | Male | Adult | Fresh | Gran Canaria | Live | Undetermined |
| **ANIMAL 4** | CET 907 | (SA90/18) | *Stenella coeruleoalba* | Male | Calf | Fresh | Gran Canaria | Dead | Pathology associated with significant loss of nutritional status |
| **ANIMAL 5** | CET 920 | (SA220/18) | *Globicephala macrorhynchus* | Male | Juvenile | Fresh | Gran Canaria | Live | Pathology associated with significant loss of nutritional status |
| **ANIMAL 6** | CET 930 | (SA256/18) | *Delphinus delphis* | Male | Adult | Fresh | Fuerteventura | Dead | Interaction with fishing activities |
| **ANIMAL 7 (*)** | CET 933 | (SA336/18) | *Stenella coeruleoalba* | Male | Subadult | Fresh | Gran Canaria | Live | Pathology associated with significant loss of nutritional status |
| **ANIMAL 8 (‡)** | CET 935 |  | *Stenella coeruleoalba* | Male | Juvenile | Alive | Gran Canaria | Live | Live animal |
| **ANIMAL 9** | CET 999 | (SA279/19) | *Stenella coeruleoalba* | Female | Newborn | Very fresh | Gran Canaria | Live | Neonatal/Perinatal pathology |

Detail of the data of the cetaceans participating in this study. (*) Frozen animals. (†) Animal used for anatomical research purposes. (‡) Animal released back into the sea.

**Supplemental table 3. Summary of the immunohistochemical methodology used in this study.**

| **ANTIGEN RETRIEVAL** | **SERUM** | **SOURCE** | **DILUTION** | **PRIMARY ANTIBODY** | **SOURCE** | **HOST** | **TYPE** | **DILUTION** | **SECONDARY ANTIBODY** | **SOURCE** | **DILUTION** |
| --- | --- | --- | --- | --- | --- | --- | --- | --- | --- | --- | --- |
| Citrate buffer (*) | Swine serum (‡) | Dako (§) | 10% (\|\|) | Myoglobin (#) | Abcam (§§) | Rabbit | Polyclonal | 1 in 200 (\|\|\|\|) | Polyclonal Swine Anti-Rabbit Immunoglobulins (‡‡‡) | Dako (§) | 1 in 200 (§§§) |
| Citrate buffer (*) | Swine serum (‡) | Dako (§) | 10% (\|\|) | Fibrinogen (**) | Abcam (§§) | Rabbit | Polyclonal | 1 in 50 (##) | Polyclonal Swine Anti-Rabbit Immunoglobulins (‡‡‡) | Dako (§) | 1 in 200 (§§§) |
| Citrate buffer (†) | Swine serum (‡) | Dako (§) | 10% (\|\|) | Troponin I (††) | Abcam (§§) | Rabbit | Polyclonal | 1 in 25 (***) | Polyclonal Swine Anti-Rabbit Immunoglobulins (‡‡‡) | Dako (§) | 1 in 200 (§§§) |
| Citrate buffer (†) | Swine serum (‡) | Dako (§) | 10% (\|\|) | Troponin C (‡‡) | Abcam (§§) | Rabbit | Monoclonal | 1 in 250 (†††) | Polyclonal Swine Anti-Rabbit Immunoglobulins (‡‡‡) | Dako (§) | 1 in 100 (\|\|\|\|\|\|) |

Detail of the immunohistochemical protocol used in this study. (*) Citrate buffer, pH 6.0, 7 minutes at 96ºC. (†) Citrate buffer, pH 6.0, 20 minutes at 100ºC. (‡) Dako Swine serum (Normal) (X090110-8). (§) Dako (Glostrup, Denmark). (||) Dilution of 10 µl of serum in 90 µl of PBS and incubated in a humidity chamber for half an hour. (#) Anti-Myoglobin antibody (ab187506). (**) Anti-Fibrinogen antibody (ab34269). (††) Anti-Cardiac Troponin I antibody (ab47003). (‡‡) Anti-Cardiac Troponin C antibody (ab137130). (§§) Abcam (Cambridge, United Kingdom). (||||) Dilution of 1 µl of antibody in 199 µl of serum at 1% in PBS and is incubated in a humidity chamber for at least 18 hours, inside the refrigerator. (##) Dilution of 1 µl of antibody in 49 µl of serum at 1% in PBS and incubated in a humidity chamber for at least 18 hours, inside the refrigerator. (***) Dilution of 1 µl of antibody in 24 µl of serum at 1% in PBS and incubated in a humidity chamber for at least 18 hours, inside the refrigerator. (†††) Dilution of 1 µl of antibody in 249 µl of serum at 1% in PBS and incubated in a humidity chamber for at least 18 hours, inside the refrigerator. (‡‡‡) Dako Polyclonal Swine Anti-Rabbit Immunoglobulins/Biotinylated (E035301-2). (§§§) Dilution of 1 µl of antibody in 199 µl of serum at 1% in PBS and incubated in a humidity chamber for half an hour. (||||||) Dilution of 1 µl of antibody in 99 µl of serum at 1% in PBS and incubated in a humidity chamber for half an hour.
